# Genome sequences and annotation of two urinary isolates of *E. coli*

**DOI:** 10.1186/s40793-016-0202-6

**Published:** 2016-10-12

**Authors:** Travis K. Price, Arya Mehrtash, Laurynas Kalesinskas, Kema Malki, Evann E. Hilt, Catherine Putonti, Alan J. Wolfe

**Affiliations:** 1 Department of Microbiology and Immunology, Stritch School of Medicine, Health Sciences Division, Loyola University Chicago, 2160 South First Avenue, Maywood, IL 60153 USA; 2 Bioinformatics Program, Loyola University Chicago, Chicago, IL USA; 3 Department of Biology, Loyola University Chicago, Chicago, IL USA; 4 Department of Computer Science, Loyola University Chicago, Chicago, IL USA

**Keywords:** *Enterobacteriaceae*, *Escherichia coli*, UPEC, Urinary tract infection, Bladder, Lower urinary tract symptoms

## Abstract

The genus *Escherichia* includes pathogens and commensals. Bladder infections (cystitis) result most often from colonization of the bladder by uropathogenic *E. coli* strains. In contrast, a poorly defined condition called asymptomatic bacteriuria results from colonization of the bladder with *E. coli* strains without symptoms. As part of an on-going attempt to identify and characterize the newly discovered female urinary microbiota, we report the genome sequences and annotation of two urinary isolates of *E. coli*: one (E78) was isolated from a female patient who self-reported cystitis; the other (E75) was isolated from a female patient who reported that she did not have symptoms of cystitis. Whereas strain E75 is most closely related to an avian extraintestinal pathogen, strain E78 is a member of a clade that includes extraintestinal strains often found in the human bladder. Both genomes are uncommonly rich in prophages.

## Introduction

Clinicians typically equate the presence of bacteria in urine with infection, or, less commonly, an ill-defined phenomenon termed “asymptomatic bacteriuria.” These and other existing concepts are based on the long-held “sterile urine” paradigm. Recently, however, bacterial communities (microbiota) have been discovered in the female bladder [1–9]. Thus, the “sterile urine” paradigm is no longer valid.

In an effort to provide a comprehensive view of the newly discovered female urinary microbiota, we have established an Enhanced Quantitative Urine Culture protocol. This enhanced culture protocol isolates bacteria from 75 to 90 % of urine samples deemed ‘no growth’ by the standard clinical microbiology urine culture method [4, 7, 10]. We have begun to the sequence and annotate the genomes of these isolated bacteria.

Here, we report the full genome sequences and annotations of two of those bacteria, *Escherichia coli* strains E75 and E78 isolated from female patients pursuing urogynecologic clinical care. Strain E75 was isolated from a patient who thought that she did not have a urinary tract infection, while E78 was isolated from a patient who thought that she did. The strains were sub-cultured to purity and then identified as *E. coli* by Matrix-Assisted Laser Desorption/Ionization-Time-of-Flight Mass Spectrometry [10]. Strain E75 is most closely related to APEC O1, an avian extraintestinal pathogen. In contrast, strain E78 is a member of a clade that includes extraintestinal strains often associated with the human bladder, including uropathogenic strains UTI89 and J89 and asymptomatic bacteriuric strain ABU83972. Both genomes are uncommonly rich in prophages.

## Organism information

### Classification and features

*Escherichia coli* is a non-sporulating, Gram-negative, rod shaped bacterium. It is a facultative anaerobe found commonly in the environment and the lower intestines of mammals and other endotherms. Extra-intestinal strains can colonize other organs, including the urinary bladder. Most *E. coli* strains are harmless constituents of the normal microbiota, but others cause disease. For example, uropathogenic *E. coli* is the major case of urinary tract infections in humans; other *E. coli* strains colonize the bladder without causing symptoms, a condition called asymptomatic bacteriuria.

**Fig. 1:**
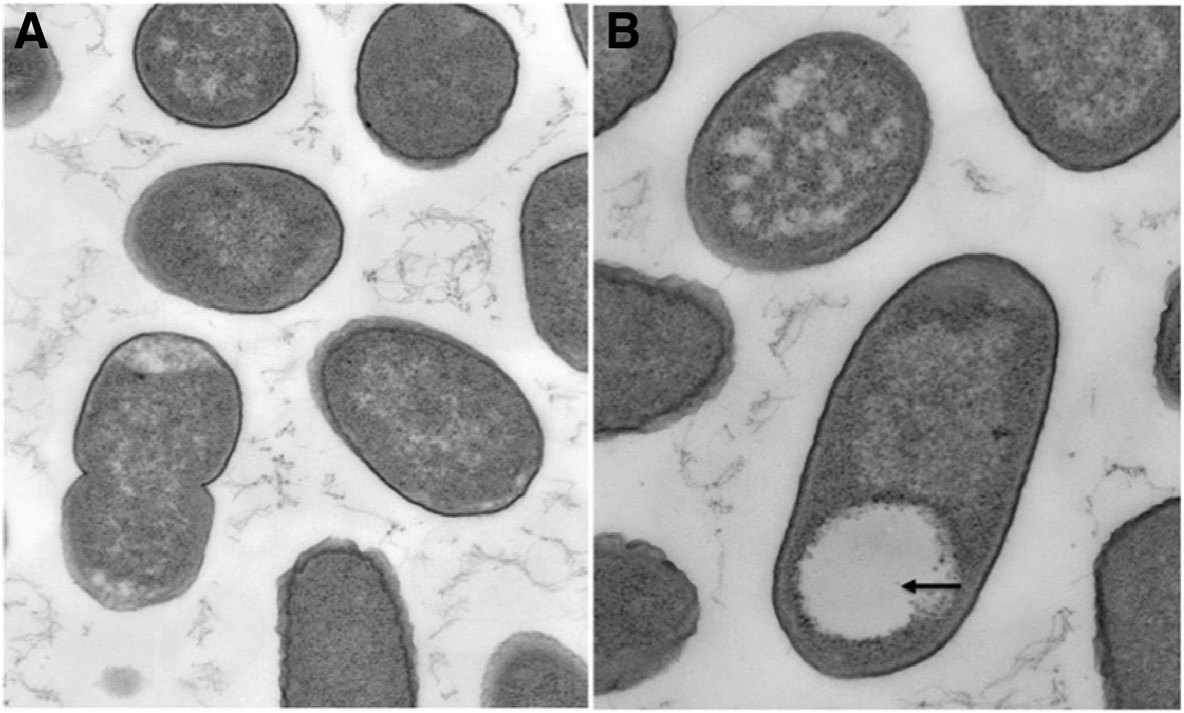
Transmission Electron Microscopy Images of E78 (**a**) and E75 (**b**). E75 tended to have electron poor intracellular inclusions (*black arrow*)

Transmission electron microscopy images were generated for both E75 and E78 (Fig. 1). Cell pellets were fixed with 0.1 % Ruthenium Red en bloc with sequential gluteraldehyde and osmium tetroxide fixation steps. These fixed samples were dehydrated with Ethanol and embedded in Resin. Ultrathin sections of 80 nm were mounted on copper grids, post-stained with uranyl acetate and lead citrate and observed in a Hitachi H-600 transmission electron microscope at 75 kV. Films were taken, negatives developed and scanned via a Microtek i800 film scanner. PhotoShop was used to convert negatives to positive images and adjust for brightness and contrast. The transmission electron micrographs revealed the typical *E. coli* rod-shape morphology. Strain E75 tended to possess electron poor intracellular inclusions (Fig. 1b, *black arrow*). The general features of *E. coli* strains E75 and E78 are presented in Table 1.

**Table 1:** Classification and general features of *E. coli* strains E75 and E78

MIGS ID	Property	Term	Evidence code^a^
	Classification	Domain *Bacteria*	TAS [31]
		Phylum *Proteobacteria*	TAS [32]
		Class *Gammaproteobacteria*	TAS [33, 34]
		Order *Enterobacteriales*	TAS [35]
		Family *Enterobacteriaceae*	TAS [36, 37]
		Genus *Escherichia*	TAS [37, 38]
		Species *Escherichia coli*	TAS [37, 38]
		Strain: E75 and E78	
	Gram stain	Negative	TAS [39]
	Cell shape	Rod	TAS [39]
	Motility	Motile	TAS [39]
	Sporulation	Non-spore former	NAS
	Temperature range	7–46 °C	NAS
	Optimum temperature	37 °C	IDA
	pH range; Optimum	4.4–9.0; 6–7	IDA
	Carbon source	Not determined, strains grown in complex medium	NAS
MIGS-6	Habitat	Human female bladder	NAS
MIGS-6.3	Salinity	0.5 % (w/v)	NAS
MIGS-22	Oxygen requirement	Facultative anaerobe	TAS [39]
MIGS-15	Biotic relationship	Human specimen	NAS
MIGS-14	Pathogenicity	Non-pathogen (E75) Suspected pathogen (E78)	NAS
MIGS-4	Geographic location	Maywood, IL USA	NAS
MIGS-5	Sample collection	E75 (9/14/2014); E78 (9/25/2014)	
MIGS-4.1	Latitude	41.8811° N	
MIGS-4.2	Longitude	87.8433° W	
MIGS-4.4	Altitude	623 ft	

^a^Evidence codes—IDA: Inferred from Direct Assay; TAS: Traceable Author Statement (i.e., a direct report exists in the literature); NAS: Non-traceable Author Statement (i.e., not directly observed for the living, isolated sample, but based on a generally accepted property for the species, or anecdotal evidence). These evidence codes are from the Gene Ontology project [40]

*E. coli* strains E75 and E78 were isolated from patients who sought clinical care at Loyola University Medical Center’s Female Pelvic Medicine and Reconstructive Surgery center in September 2014. Patients were asked the question: Do you feel that you have a urinary tract infection? E75 was isolated from a patient who answered ‘no,’ whereas E78 was isolated from a patient who answered ‘yes.’ Both patients were white, post-menopausal women seeking care for Pelvic Organ Prolapse. Neither patient was taking antibiotics; both were using daily vaginal estrogen supplement. The UTI Symptoms Assessment Questionnaire was used to characterize the degree of severity and bother of the patients’ symptoms [11]. Both *E. coli* strains were identified at >100,000 colony forming units per milliliter, using an Expanded Spectrum version of the Enhanced Quantitative Urine Culture protocol [10]. After they were sub-cultured to purity, Matrix-Assisted Laser Desorption/Ionization-Time-of-Flight Mass Spectrometry was used to confidently identify them as *E. coli*. For E75, the identification score was 2.530; for E78, the score was 2.265. No other microbes were detected in the urine sample containing strain E75. In the urine sample containing strain E78, *Alloscardovia omnicolens* (10 colony forming units per milliliter) and *Lactobacillus rhamnosus* (10 colony forming units per milliliter) were also detected.

**Fig. 2:**
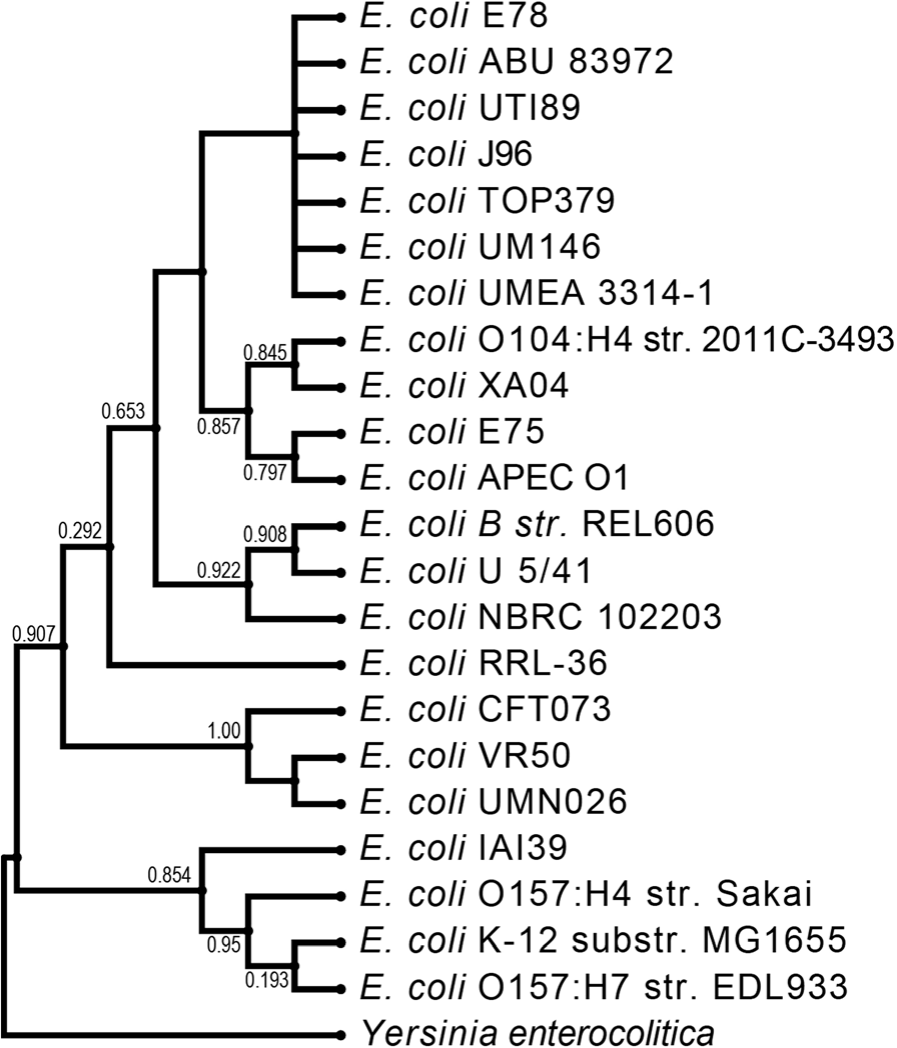
Phylogenetic tree based on 16S rRNA sequences. The alignment length was 1189 bp. Sequences were retrieved from NCBI and aligned using Muscle. The tree was generated by FastTree using the GTR model. Support values are shown for branches leading to the placement of the two bladder isolates presented here

Figure 2 shows a phylogenetic tree of the 16S rRNA sequences. 16S rRNA gene sequences include *Yersinia enterocolitica* (NR_104903), *E. coli* IAI39 (NC_011750), *E. coli* O157:H7 str. Sakai (NR_074891), *E. coli* K-12 substr. MG1655 (NR_102804), *E. coli* O157:H7 str. EDL933 (AE005174), *E. coli* CFT073 (AE014075), *E. coli* VR50 (CP011134), *E. coli* UMN026 (NC_011751), *E. coli* RRL-36 (JQ398845), *E. coli* NBRC 102203 (NR_114042), *E. coli* U 5/41 (NR_024570), *E. coli* B str. REL606 (CP000819), *E. coli* O104:H4 str. 2011C-3493 (NC_018658), *E. coli* XA04 (KR080744), *E. coli* APEC O1 (CP000468), *E. coli* E75, *E. coli* E78, *E. coli* J96 (ALIN02000018), *E. coli* TOP379 149 (AOQB01000139), *E. coli* UMEA 3314-1 (AWDE010000004), *E. coli* UTI89 (CP000243), *E. coli* ABU 83972 (CP001671), and *E. coli* UM146 (CP002167). *E. coli* genome sequences typically include seven copies [12].

## Genome sequencing information

### Genome project history

The sequencing and quality assurance was performed at the Loyola Genome Facility at Loyola University Chicago, Maywood, IL, USA. The assemblies and finishing were done at the Lakeshore Campus of Loyola University Chicago, Chicago, IL, USA. Functional annotation was produced by the RAST service [13] and in-house scripts for COG classification [14]. Table 2 presents the project information and its association with MIGSversion2.0compliance [15].

**Table 2:** Project information

MIGS ID	Property	E75 Term	E78 Term
MIGS 31	Finishing quality	High quality draft	High quality draft
MIGS-28	Libraries used	Paired-end library of 150 bp	Paired-end library of 150 bp
MIGS 29	Sequencing platforms	Illumina MiSeq	Illumina MiSeq
MIGS 31.2	Fold coverage	51-431×	53-30724×
MIGS 30	Assemblers	Velvet	Velvet
MIGS 32	Gene calling method	GLIMMER	GLIMMER
	Locus tag		
	Genbank ID	LXGO00000000	LXQH00000000
	GenBank Date of Release	May 9, 2016	May 9, 2016
	GOLD ID		
	BIOPROJECT	PRJNA316969	PRJNA316969
MIGS 13	Source Material Identifier		
	Project relevance	Human commensal	Human pathogen

### Growth conditions and genomic DNA preparation

*E. coli* strains E75 and E78 were isolated from transurethral catheterized urine specimens of adult women with urinary symptoms [10] using a Expanded Spectrum version of the previously described Enhanced Quantitative Urine Culture protocol [4]. Three urine volumes (1 μL, 10 μL, and 100 μL) of each urine sample was spread quantitatively (i.e., pinwheel streak) onto t5% sheep blood (BD BBL™ Prepared Plated Media, Cockeysville, MD), Chocolate, and Colistin Naladixic Acid agars (BD BBL™ Prepared Plated Media) and incubated in 5 % CO_2_ at 35 °C for 48 h; 5 % sheep blood and MacConkey (BD BBL™ Prepared Plated Media) agars incubated aerobically at 35 °C for 48 h; two CDC Anaerobic 5 % sheep blood agars (BD BBL™ Prepared Plated Media) incubated in either Microaerophilic Campy gas mixture (5 % O_2_, 10 % CO_2_, 85 % N), or anaerobically at 35 °C for 48 h. All agars were documented for growth (i.e., for morphologies and colony forming units per milliliter) at 24 and 48 h. Each distinct colony morphology was sub-cultured at 48 h to obtain pure culture for microbial identification.

Microbial identification was determined using a Matrix-Assisted Laser Desorption/Ionization-Time-of-Flight Mass Spectrometer (Bruker Daltonics, Billerica, MA) as described [4]. Pure cultures were stored at -80 °C in a 2 ml CryoSaver *Brucella* Broth with 10 % Glycerol, no beads, Cryovial, for preservation (Hardy Diagnostics). For genome extraction and sequencing, the preserved pure culture isolates were grown on 5 % sheep blood agar under aerobic conditions at 35 °C for 24 h.

Genomic DNA extraction was performed using a phenol-chloroform extraction protocol. Briefly, cells were resuspended in 0.5 mL DNA Extraction Buffer (20 mM Tris-Cl, 2 mM EDTA, 1.2 % Triton X-100, pH 8) followed by addition of 50uL Lysozyme (20 mg/mL), 30uL Mutanolysin, and 5uL RNase (10 mg/mL). After a 1 h incubation at 37 °C, 80uL 10 % SDS, and 20uL Proteinase K were added followed by a 2 h incubation at 55 °C. 210uL of 6 M NaCl and 700uL phenol-chloroform were then added. After a 30-min incubation with rotation, the solutions were centrifuged at 13,500 RPM for 10 min, and the aqueous phase was extracted. An equivalent volume of Isopropanol was then added, and solution was centrifuged at 13,500 RPM for 10 min after a 10-min incubation. The supernatant was decanted and the DNA pellet was precipitated using 600uL 70 % Ethanol. Following ethanol evaporation, the DNA pellet was resuspended in Tris-EDTA and stored at -20 °C.

### Genome sequencing and assembly

DNA samples were diluted in water to a concentration of 0.2 ng/ul as measured by a fluorometric-based method (Life Technologies, Carlsbad, CA) and 5 ul was used to obtain a total of 1 ng of input DNA. Library preparation was performed using the Nextera XT DNA Library Preparation Kit (Illumina, San Diego, CA) according to manufacturer’s instructions. The isolates were barcoded, pooled and each isolate was sequenced twice, on two separate runs, using the Illumina MiSeq platform and the MiSeq Reagent Kit v2 (300-cycles) to produce 150 bp paired-end reads. Sequencing reads were parsed into individual folders according to the respective barcodes.

Sequence assembly was conducted using Velvet [16] (Table 2). The tool VelvetOptimiser was used to determine the best hash length; 99 was used in the two assemblies performed here. The scaffolding software SSPACE [17] was utilized for scaffold finishing. The genome of strain E75 was assembled into 463 contigs. The genome of strain E78 was assembled into 62 contigs. To confirm that the contigs were *Enterobacteriaceae* (i.e. not the result of contamination), each contig was BLASTed locally against all publicly available bacterial genomes (obtained from NCBI). Coverage across all contigs was on average 50.95-431.17X (for E75) and 52.59-30,723.61X (for E78). The high coverage observed within the E78 sequencing is the result of two contigs, one 4083 bp in length (coverage 72,734X) and the other 2113 bp in length (99,530X). Assembly was repeated using the SPAdes assembler [18], given its recent success in producing full plasmid sequences [19]. Two plasmids were identified by the SPAdes assembler with high coverage. Querying these two contigs against the GenBank nr/nt database revealed sequence homology to the annotated *E. coli* plasmids p2PCN033 (GenBank: CP006634) and pVR50G (GenBank: CP011141) (among other *E. coli* plasmids), respectively. These two plasmids are listed in Table 3 as Plasmid pE78.1 and Plasmid pE78.2, respectively. All 62 E78 contigs were also assessed for putative plasmid sequences using PlasmidFinder [20]. While PlasmidFinder recognized pE78.2, it did not detect pE78.1. The complete genome of the E78 chromosome is thus represented within 60 contigs (mean coverage 272×).

**Table 3:** Summary of genomes: two chromosomes and two plasmids

Label	Size (bp)	Topology	INSDC identifier
Chromosome E75	5,032,328	Circular	LXGO00000000
Chromosome E78	5,021,201	Circular	LXQH00000000
Plasmid pE78.1	4083	Circular	LXQH00000000
Plasmid pE78.2	2113	Circular	LXQH00000000

### Genome annotation

Genes were identified using GLIMMER using the g3-from-scratch.csh script included in the package [21] The predicted CDSs were translated using the transeq script within the EMBOSS suite [22]. rRNA genes were identified by RNAmmer [23] using the parameter set to identify bacterial rRNA sequences. The program tRNA-Scan [24] identified tRNA sequences, using the parameter for bacterial tRNAs. Trans-membrane proteins were identified using TMHMM with standard parameters [25]. SignalP [26] predicted signal peptides. All CDSs were queried (blastp) locally against the COG sequence dataset ([14]) and assigned based upon their sequence homologies. CRISPR elements were detected through CRISPR-db [27]. Genes with Pfam domains were ascertained via searches of the Pfam database (E-value threshold 1.0) [28].

## Genome properties

Tables 4 and 5 include the summaries of the properties and statistics of each genome. Sequencing of the E78 isolate identified two plasmids (Table 3); the E75 isolate did not contain any identifiable plasmid sequences. The E75 and E78 chromosomes are similar in length and GC content: E75 is 5,032,328 bp (GC content 50.4 %), while E78 is 5,021,201 bp (GC content 50.3 %). The genomes for E75 and E78 are predicted to include 4587 and 4743 protein coding genes, respectively. A similar coding density is observed within the two genomes. The 85 RNA genes identified within the E75 genome include 78 tRNAs and 7 rRNAs. The E78 genome encodes for more RNA genes: 83 tRNAs and 13 rRNAs. The scaffolds of E75 and E78 are only annotated as having a single 16S rRNA gene, an underestimation due to recognized challenges of assembling sequences containing genes with multiple copies such as the rRNA genes [29] Thus, we fully expect that the E75 and E78 genomes harbor rRNA gene numbers on par with the genus.

**Table 4:** Genome statistics

Strain	E75		E78	
Attribute	Value	% of Total^a^	Value	% of Total^a^
Genome size (bp)	5,032,328	100.00	5,021,201	100.00
DNA coding (bp)	4,466,253	88.75	4,348,152	86.60
DNA G + C (bp)	2,537,751	50.43	2,525,290	50.29
DNA scaffolds	463	na	60	na
Total genes	4666	100.00	4839	100.00
Protein coding genes	4581	98.18	4743	98.02
RNA genes	85	1.82	96	1.98
Pseudo genes	0	0	0	0
Genes in internal clusters	na	na	na	na
Genes with function prediction	3290	70.51	3401	70.28
Genes assigned to COGs	3496	74.92	3603	74.46
Genes with Pfam domains	2067	44.30	2233	46.15
Genes with signal peptides	361	7.74	374	7.73
Genes with transmembrane helices	1083	23.21	1114	23.02
CRISPR repeats	6		5	

^a^The total is based on either the size of the genome in base pairs or the total number of protein coding genes in the annotated genome

**Table 5:** Number of genes associated with general COG functional categories

Strain	E75		E78		
Code	Value	%age	Value	%age	Description
J	237	5.16	240	5.06	Translation, ribosomal structure and biogenesis
A	2	0.04	2	0.04	RNA processing and modification
K	242	5.28	258	5.44	Transcription
L	151	3.29	152	3.21	Replication, recombination and repair
B	0	0	0	0	Chromatin structure and dynamics
D	44	0.96	43	0.91	Cell cycle control, Cell division, chromosome partitioning
V	85	1.85	82	1.73	Defense mechanisms
T	158	3.45	160	3.37	Signal transduction mechanisms
M	238	5.19	241	5.05	Cell wall/membrane biogenesis
N	89	1.94	95	2.00	Cell motility
U	52	1.13	50	1.05	Intracellular trafficking and secretion
O	149	3.25	149	3.14	Posttranslational modification, protein turnover, chaperones
C	266	5.80	278	5.86	Energy production and conversion
G	366	7.98	386	8.14	Carbohydrate transport and metabolism
E	336	7.33	338	7.13	Amino acid transport and metabolism
F	104	2.27	102	2.15	Nucleotide transport and metabolism
H	167	3.64	169	3.56	Coenzyme transport and metabolism
I	115	2.51	118	2.49	Lipid transport and metabolism
P	213	4.64	212	4.47	Inorganic ion transport and metabolism
Q	53	1.16	53	1.12	Secondary metabolites biosynthesis, transport and catabolism
R	173	3.77	178	3.75	General function prediction only
S	206	4.50	202	4.26	Function unknown
-	1085	23.69	1140	24.04	Not in COGs

The total is based on the total number of protein coding genes in the genome

## Insights from the genome sequence

Although E75 was isolated from a woman who reported that she did not have symptoms of cystitis, its genome encodes proteins associated with *E. coli* pathogenesis, including the P pilus, RTX toxin, and α-fimbriae. These genes were not found in E78. While the E75 strain did not include plasmid sequences, genome sequencing of the E78 isolate contained two. Plasmid pE78.2 was nearly identical (one mismatch) to the *E. coli* plasmid pVR50G, collected from urine obtained from an individual with asymptomatic bacteriuria [30].

Both genomes included a number of prophages. Each prophage sequence within the genomes was BLASTed (blastx) to the nr/nt database revealing numerous hits to phage sequences annotated as infecting *Escherichia* spp. Annotations within the genomes of the temperate phages Lambda and P4 were identified most frequently within the E75 and E78 genomes, respectively. Table 6 lists the statistics of this search. The vast majority of the hits were to phages annotated as infectious for *Escherichia*, *Salmonella*, and/or *Shigella* spp. Nevertheless, prophage sequences for both temperate as well as lytic phages were identified. The abundance of prophage sequences within these two genomes exceeds that previously identified in *E. coli* genomes.

**Table 6:** Predicted sequences of phage origin and putative origin

	E75	E78
Number of predicted phage CDSs	112	112
Exhibit no sequence homology to GenBank	4	10
Species with most hits (# hits)	*Enterobacteria* phage lambda (19)	Bacteriophage P4 (10)

Sequence homologies determined via blastx

## Conclusions

The genome of E75, isolated from a woman who reported no symptoms of cystitis, is more closely related to the avian extraintestinal pathogen APEC 01. The genome of E75, isolated from a woman who reported cystitis symptoms, resides in a clade populated by human extra-intestinal strains that are either uropathogenic or asymptomatic bacteriuric. Both genomes contain an unusually large number of prophage sequences.
